# A nationwide survey on clinical neurophysiology education in Italian schools of specialization in neurology

**DOI:** 10.1007/s10072-021-05641-0

**Published:** 2021-12-09

**Authors:** Tommaso Bocci, Laura Campiglio, Vincenzo Silani, Alfredo Berardelli, Alberto Priori

**Affiliations:** 1 Clinical Neurology Unit, ASST Santi Paolo & Carlo and Department of Health Sciences, University of Milan, Via Antonio di Rudinì 8, 20100 Milano, Italy; 2 Aldo Ravelli” Center for Neurotechnology and Experimental Brain Therapeutics, University of Milan, Milan, Italy; 3 Department of Neurology, Stroke Unit and Laboratory Neuroscience, “Istituto Auxologico Italiano”, IRCCS, Department of Pathophysiology and Transplantation “Dino Ferrari Center”, University of Milan, Milan, Italy; 4 Department of Human Neurosciences and IRCCS Neuromed Institute, Sapienza University of Rome, Rome, Italy

**Keywords:** Medical education, Clinical neurophysiology, Specialization in neurology, Training in neurophysiology

## Abstract

**Introduction:**

Clinical neurophysiology deals with nervous system functions assessed with electrophysiological and ultrasound-based imaging techniques. Even though the need for highly specialized neurophysiologists has increased, residency training rarely takes today’s requirements into account. This study aimed to snapshot the neurophysiological training provided by Italian specialization schools in neurology.

**Methods:**

A single-page web-based survey comprising 13 multiple-choice categorical and interval scale questions was sent via e-mail to neurology specialization school directors. The survey addressed the programs’ structural neurophysiology organization, time dedicated to each clinical neurophysiology subspecialty, and descriptors assessing the discipline’s importance (e.g., residents who attempted residential courses, gained certifications, or awards gained).

**Results:**

The most studied neurophysiological techniques were electroencephalography (EEG) and electromyography (EMG). Most specialization schools devoted less than 3 months each to multimodal evoked potentials (EPs), ultrasound sonography (US), and intra-operative monitoring. Of the 35 specialization schools surveyed, 77.1% reported that four students, or fewer, participated in the Italian Society of Clinical Neurophysiology Examination in Neurophysiology. Of the 35 specialization centers surveyed, 11.4% declared that the final evaluation required students to discuss a neurophysiological test.

**Discussion:**

Our survey underlined the poorly standardized technical requirements in postgraduate neurology specialization schools, wide variability among training programs, and limited training on multi-modal evoked potentials, intraoperative monitoring, and sonography. These findings underline the need to reappraise and improve educational and training standards for clinical neurophysiology during postgraduate specialization schools in neurology with an international perspective.

## Introduction

Clinical neurophysiology (CN) according to the International Federation of Clinical Neurophysiology (IFCN) is a “medical specialty concerned with function and dysfunction of the nervous system caused by disorders of the brain, spinal cord, peripheral nerve and muscle, using physiological and imaging techniques to measure nervous system activity” (http://www.ifcn.info).

Conventional neurophysiological techniques include two main areas: studies investigating brain activity: electroencephalography (EEG) and those investigating the peripheral nervous system: nerve conduction studies (NCS) and electromyography (EMG). In the modern era, neurophysiological methods have greatly expanded to include techniques traditionally used in daily clinical practice (EEG, NCS, EMG, evoked potential studies, polysomnography and assessment of sleep disorders, vascular sonography), as well as emerging diagnostic methods, including nerve sonography, vagal nerve stimulation (VNS) for epilepsy, exercise testing for muscle fatigue, intra-operative monitoring (IOM) and neurophysiological assessment of movement disorders [1–3].

In our experience, during their stay in the neurology unit, each hospitalized patient undergoes at least one neurophysiological test. Clinicians frequently prescribe neurophysiological investigations also for neurological outpatient diagnostic assessment. Even though no comprehensive national data specify the number of outpatient neurophysiological tests conducted per year in Italy, data are available for some regions. For instance, in Lombardy in 2017, the national health system provided more than 2 million neurological visits and tests, corresponding to euro 35 million in revenue. Neurophysiological tests account for more than a half of this revenue approaching 18 million euros (Table 1).

**Table 1:** Outpatient neurology service in Lombardy in 2017. *NF*, neurophysiology. *In Lombardy, each single nerve and muscle is counted for administrative payment

	Exam/visit	Amount				Revenue			
		*N*		%		Euros		%	
Not NF	Neurological visit	538,920.00	792,751.00	25.44%	37.42%	10,954,484.80	13,062,728.90	32.46%	38.71%
	Neuropsychological tests	186,357.00		8.80%		1,462,310.60		4.33%	
	Botulinum toxin injection	67,128.00		3.17%		637,716.00		1.89%	
	Spinal cord stimulator programming	346.00		0.02%		8217.50		0.02%	
NF	Electroencephalogram	68,857.00	1,257,074.00	3.25%	59.33%	1,774,417.30	20,686,483.65	5.26%	61.29%
	Nerve conductions/electromyography*	1,106,376.00		52.22%		11,685,944.40		34.63%	
	EEG/poligraphy	5982.00		0.28%		418,693.30		1.24%	
	Multimodal evoked potentials	39,888.00		1.88%		1,455,459.64		4.31%	
	Polysomnography/actigraphy	35,089.00		1.66%		5,281,673.61		15.65%	
	NF tests for autonomic functions	882.00		0.04%		70,295.40		0.21%	
Total	Total	2,049,825.00				33,749,212.55			

Adding to the problem concerning the many neurophysiological tests neurologists need to be familiar with, in the past few years, many reports using neurophysiological techniques as therapeutic tools appeared. Published papers now increasingly recognize the emerging field of non-invasive brain stimulation (including repetitive magnetic stimulation, rTMS, and transcranial direct current stimulation, tDCS) as safe treatments for several neurological and neuropsychiatric diseases [4–7], ranging from chronic pain and movement disorders, to drug-resistant depression and cognitive enhancement [6–9]. As treatment options for movement disorders, invasive brain stimulation has rapidly evolved, with new neurosurgical methods, anatomical targets and neurophysiological markers [10, 11].

Despite the importance of CN in neurological clinical practice, few published data refer to education in this field during postgraduate neurological training demonstrating wide variability in different countries. In 21/32 (66%) of European countries, CN belongs in the neurology residency program [6]. Conversely in Spain, Portugal, the UK, Finland, Sweden, and Norway, CN is considered a different medical specialty. In the USA, CN is a subspecialty: neurologists, child neurologists, or psychiatrists can acquire CN certification usually through a 1-year fellowship [12, 13]. Before 2017, in Italy, postgraduate medical students studied CN as an independent 5-year residency program: during their first 2 years training, residents usually acquired general neurological practice skills, whereas in the last 3 years, they focused on neurophysiological techniques among other subspecialties. After 2017, CN was integrated in a 4-year neurology residency program. The rapidly expanding neurological sciences and the increased pressure in each subspecialty area on the program led Italian neurology residents to have an enormous amount of information to learn. No published study has evaluated the educational level in CN for neurology residents in Italy but the European Training Requirements for Neurology of the European Board and Section of Neurology (U.E.M.S.) are quite demanding. Knowing more about CN training in Italian postgraduate specialization schools in neurology during residency would help plan strategies for updating them to fit in with today’s neurologists’ increasingly technical needs.

Our study aimed to conduct a nationwide web-survey to snapshot the neurophysiological training provided by Italian specialization schools in neurology.

## Materials and methods

We designed a single-page, Internet-based survey comprising 13 multiple choice categorical and interval scale questions. Italian neurology specialization school directors were contacted via e-mail and invited to complete the online form. The survey addressed the following questions: geographical location of the specialization school and structural organizations in neurophysiology; time dedicated to each CN subspecialty; indirect signs assessing the discipline’s importance (number of residents who attempted extra residential courses, gained certification or obtained recognitions; CN test assessed during the final examination). The full survey is available as supplemental material. Data were segregated by responses and each item was assessed with descriptive statistics. The survey was available online from 1st March to 30th April 2021 for a total 61 days.

## Results

**Fig. 1:**
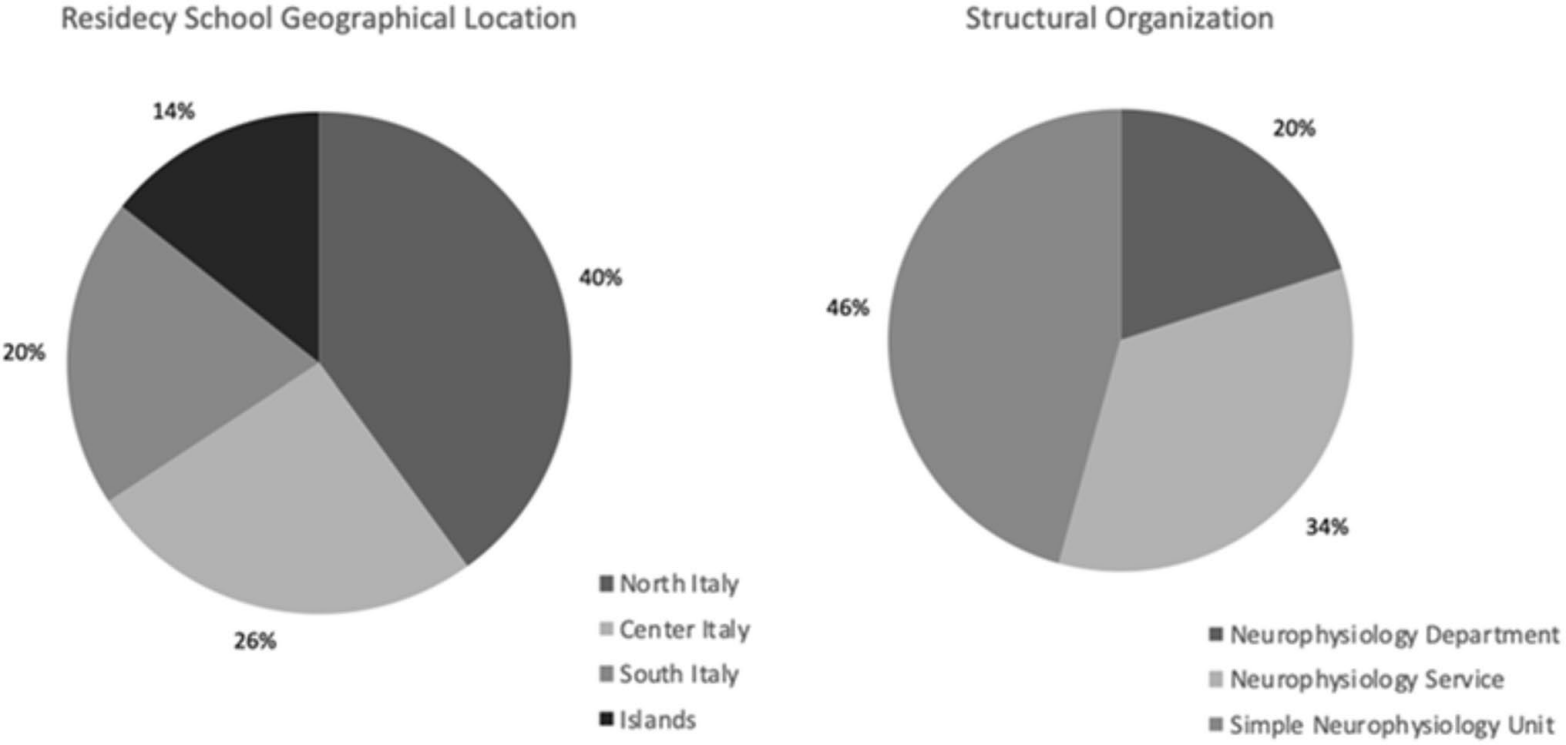
Geographical localization of the schools enrolled

**Fig. 2:**
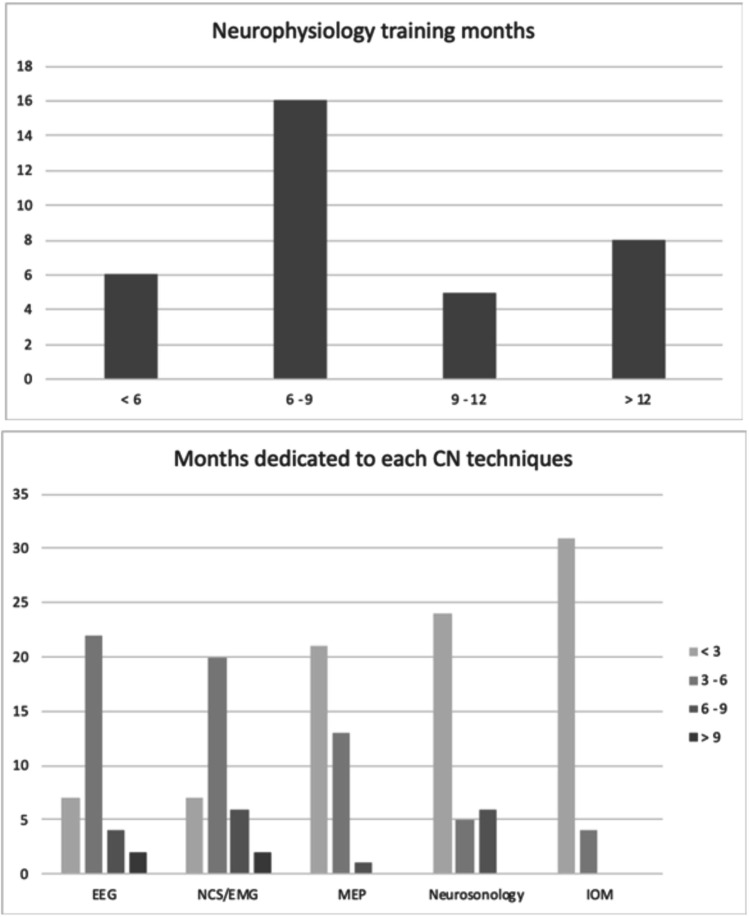
Histograms showing the cumulative time, in months, dedicated to postgraduate training in neurophysiology (at the top) and the time spent for each technique (EEG, electroencephalography; NCS/EMG, nerve conduction studies/electromyography; MEP, multimodal evoked potentials; IOM, intra-operative monitoring)

Of the 42 Italian schools of specialization in neurology contacted, 35 (83.3%) answered. Less than half (40%) were from Northern Italy. About two thirds of the centers had a Unit or a Section of CN, autonomous and formally separated from the Unit of Neurology (Fig. 1). Despite differences, the most studied CN techniques were EEG and EMG; the mean time spent in EEG and EMG training was 6 months, for each technique (Fig. 2). The specialization schools in neurology devoted less time to multimodal evoked potentials (EPs), ultrasound sonography (US), and intra-operative monitoring (IOM). About 60% of the interviewed centers reported less than 3 months spent for training in EPs, a percentage rising to 68.6% for US and to 88.6% for IOM techniques, including deep brain stimulation (DBS) for Parkinson’s disease (Fig. 2).

**Fig. 3:**
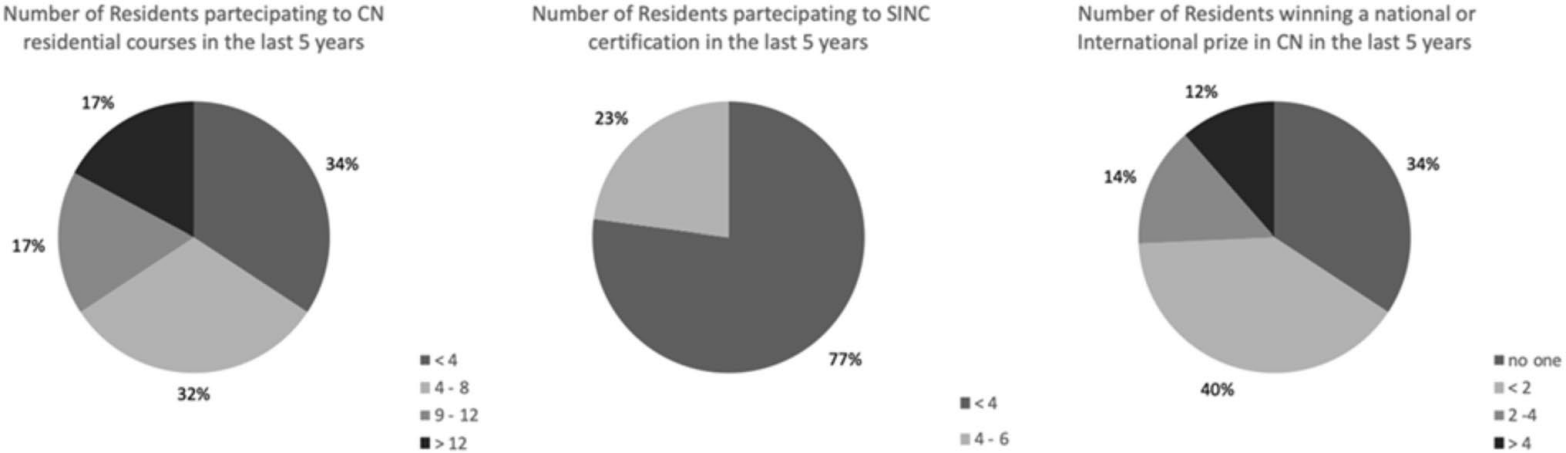
Secondary outcomes. The charts show the reported participation of residents in neurology reserved to congresses, residential courses, and to the Italian certification in clinical neurophysiology

When asked about how the specialization school during residency objectively evaluated technical requirements, 77.1% of the centers reported that only four residents, or fewer, participated in the past 5 years (2016–2021) in the Examination in Neurophysiology held by the Italian Society of Clinical Neurophysiology (“Certificazione Unica in Neurofisiologia”; Fig. 3). Only four centers (11.4%) declared that final examination during residency requires specialization students to discuss a neurophysiological test; in most schools surveyed, preparation was non-objectively assessed during the training period, without any examination (40.0%), or not assessed at all (11.4%). Accordingly, students’ interest for Congresses or Webinars on Neurophysiology, both at a national or international level, was extremely limited, with a mean of 2–4 residents per school participating in the entire period considered (2016–2021). Finally, surprisingly few residents in neurology achieved awards for studies or publications in neurophysiology fields (none in 34.3% and less than two in 40% of the cases, during the timeline 2016–2021).

## Discussion

Our national survey suggests that *curricula* in Italian specialization schools in neurology lack standardized requirements. Equally disconcerting is the wide variability among training programs (especially concerning time and neurophysiological service involved) and the limited training received on multi-modal evoked potentials, intra-operative monitoring (IOM) and sonography compared with other neurophysiological techniques. Hence, no standardized CN training is yet available and when provided its duration differs among Centers, in Italy as well as in other European countries. Our findings are hard to compare with those in other countries because similar studies are still lacking even if the European Training Requirements for Neurology of the U.E.M.S. are explicitly related to the specific requirements.

Although it has recently been proposed in the USA [14], no standardized curriculum in clinical neurophysiology, during residency in neurology, exists so far in European countries. Another critical concern during residency is external rotations, including disciplines not directly related to neurology: the conflict is to provide exposure to neighboring disciplines, while allowing sufficient time for the clinical neurophysiology core curriculum [15]. For instance, especially in the first 2 years of training, up to 6 months each year are devoted to rotation in internal medicine units.

Another reason why specialization school curricula during residency need updating is the growing need for hyper-specialized neurophysiologists due to recent advances in Telemedicine, a requirement that has gained importance during the COVID-19 pandemic outbreak [16]. Finally, another critical concern is that the recent COVID-19 pandemics have rapidly changed our knowledge about neuroinfectious diseases, prompting us to re-consider safety criteria, protocols and recording standards in clinical neurophysiology [17, 18].

Our data can hardly be compared to those described by other surveys in different countries, owing to differences in the duration of residency courses in neurology and training in neurophysiopathology; nor did other surveys evaluate training in specific technical fields, such as multimodal potentials and intra-operative monitoring. In the USA, Daniello and Weber recently developed a survey for program directors asking about confidence in neurophysiology knowledge, expressed as the percent of graduates reaching level 4 ACGME (American Council of Graduate Medical Education) milestones in EEG and EMG [12]. They reported that up to a quarter of residents may graduate not meeting level 4 ACGME milestones (i.e., the highest level of expertise in electromyography), but this American survey left unassessed the confidence in other neurophysiological techniques (e.g., vascular sonography or multimodal evoked potentials).

In Europe, Kleineberg and co-workers reported that the learning method in neurology and clinical neurophysiology significantly differs among countries, from a brief theoretical course to a defined minimum number of investigations to be performed [15]; certifications in clinical neurophysiology are often granted by different societies, with different standards, depending on the sub-specialty considered (sonography, EEG, EMG, sleep, neurovascular procedures).

The main limitation of our study is the target: each center was represented by the Director of the Neurology Unit, with no question directly reserved to neurology residents or students: the type of questionnaire administered also neglected to assess their satisfaction and opinion [19, 20]. Second, the fundamental and interplaying role of the neurophysiologist technician has not been investigated in detail: the technician can perform almost all reported examinations, apart from needle eletromyography and invasive or non-invasive brain stimulation, but the final electrophysiological diagnosis and therapeutic approaches are devoted to the physician. Based on the results of the present survey, and in line with other countries, we propose a 2-year, CN training following the residency in neurology (or neighboring disciplines). 

In conclusion, our findings underline the need to define educational and homogeneous training standards for postgraduate clinical neurophysiology in Italy and at international level.

## Data Availability

The corresponding author has full access to data and has the right to publish such data. Data will be available upon reasonable request to the corresponding author.
